# Predictors of device-related adverse events in patients with intra-aortic ballon pump or microaxial flow pump for cardiogenic shock

**DOI:** 10.1016/j.jhlto.2026.100557

**Published:** 2026-04-08

**Authors:** Zachary Provenzano, Zachary Patel, Cecil Jnawali, Roxanne Mittelstaedt, Meg Ospina, Stephanie Samani, Jacob Peller, Charlotte Marchell, Kelly Ohlrich, Brady Gunn, Michael Varrone, Kaylen Dodson, Lindsey Bull, Jennifer Hajj, Mathew J. Gregoski, Ryan J. Tedford, Walker Blanding, Jeffery McMurray, Lucas Witer, Arman Kilic, Brian A. Houston, Anthony P. Carnicelli

**Affiliations:** a Division of Cardiology, Department of Medicine, Medical University of South Carolina, Charleston, SC; b Division of Cardiothoracic Surgery, Department of Surgery, Medical University of South Carolina, Charleston, SC; c Department of Public Health Sciences, Medical University of South Carolina, Charleston, SC; d Department of Anesthesia and Perioperative Medicine, Medical University of South Carolina, Charleston, SC

**Keywords:** Advanced heart failure, Cardiogenic shock, Temporary mechanical circulatory support, Intra-aortic balloon pump, Micro-axial flow pump, Impella, Device-related adverse events

## Abstract

Device-related adverse events (DRAEs) are common with intra-aortic balloon pump (IABP) and microaxial flow pump (mAFP) in cardiogenic shock (CS), yet predictors of DRAEs are underrecognized. We performed a single-center, retrospective review of consecutive adults from 8/2021 to 8/2024 with CS and IABP or mAFP. Events were adjudicated as device-related if occurring during IABP/mAFP support or <72 hours of removal. DRAEs included bleeding, bacteremia, stroke/transient ischemic attack, vascular injury, heparin-induced thrombocytopenia, and major hemolysis. Multivariable regression was performed to identify independent predictors of DRAEs. The study population (*n* = 400) was predominantly male (71.5%) with stage D/E CS (53.7%). In total, 251 DRAEs were identified. Independent DRAE predictors included non-white race, veno-arterial extracorporeal membrane oxygenation (VA-ECMO) exposure, outside hospital device placement, and >1 device, while IABP-only was protective. Among those with mAFP, VA-ECMO exposure, outside hospital device placement, and >1 device were independent DRAE predictors. Further investigation into DRAE prevention strategies is warranted.

## Background

Intra-aortic balloon pumps (IABPs) and microaxial flow pumps (mAFPs) are the most common temporary mechanical circulatory support (tMCS) devices used in cardiogenic shock (CS).^1^ Device-related adverse events (DRAEs) on IABP and mAFP are common^2^ and are associated with poor outcomes.^3^ We aimed to identify independent predictors of DRAEs on IABP and mAFP to help inform patient selection, device management, and risk mitigation strategies.

## Methods

After Institutional Review Board approval, we conducted a single-center, retrospective analysis identifying all adult patients with IABP (inserted via arterial sheath) or mAFP (Impella CP or 5.5; Abiomed, Danvers, MA) placed for CS (defined as cardiac dysfunction and quantitative end-organ hypoperfusion) and managed at the Medical University of South Carolina from 8/2021 to 8/2024. Patients were identified using Current Procedural Terminology codes for device placement, repositioning, or removal. Patients with right-sided devices, veno-arterial extracorporeal membrane oxygenation (VA-ECMO) alone (without IABP/mAFP), devices placed for reasons other than CS, and post-cardiotomy shock were excluded.

Baseline characteristics, device data (including DRAEs), and outcomes were collected by electronic health record review. Race characterization was patient/family-reported on hospital intake. DRAE definitions were based on Shock Academic Research Consortium definitions^4^ and included bleeding, vascular injury, bacteremia, heparin-induced thrombocytopenia (HIT), stroke/transient ischemic attack (TIA), and major hemolysis (only in patients with mAFP) (Supplement Table S1). Events were adjudicated by the multidisciplinary CS team and were classified as device-related if occurring while on IABP/mAFP or <72 hours after removal.

Using IBM SPSS (IBM Corporation, Armonk, NY), continuous variables were examined with the Wilcoxon rank-sum test and categorical variables with the Chi-square or Fisher’s exact test. Univariable logistic regression was performed in the overall cohort and a sub-group with mAFPs to identify predictors of any DRAE. Variables with *p* < 0.1 were preliminarily selected for inclusion into multivariable models, then were examined for collinearity using variance inflation factor (excluded where >5). Box-Tidewell testing for linearity of the logit was completed for body mass index. Backward stepwise binary logistic regression was performed to identify independent predictors of any DRAE. Examination of Cook’s Distance (cutoff >1) and Studentized Residuals (cutoff |3|) indicated no substantial outliers. Model calibration and explanatory power were assessed using the Hosmer-Lemeshow goodness-of-fit test and Nagelkerke pseudo-R², respectively.

## Results

In total, 400 patients received 256 IABPs and 286 mAFPs during the study period (113 [28.3%] patients with >1 device; Supplement Figure S1). Baseline characteristics are described in Table 1. Overall, 161 (40.3%) patients experienced 251 DRAEs (56 [14.0%] patients with >1 DRAE), including 90 bleeding, 48 bacteremia, 13 stroke/TIA, 36 vascular injury, and 13 HIT events. Individual DRAE incidence by device exposure is shown in Supplement Table S2. Among 251 patients with mAFP exposure, 51 (20.3%) experienced major hemolysis. In-hospital mortality was 24.3%, while 26.3% underwent heart transplantation and 15.0% received durable left ventricular assist device.

**Table 1:** Baseline Characteristics

	All (*n* = 400)	DRAEs (*n* = 161)	No DRAEs (*n* = 239)	*p*-value
*Demographics*				
Male - *n (%)*	286 (71.5)	114 (70.8)	172 (72.0)	0.80
Age (years)	60.5 (19)	61 (20)	60 (19)	0.67
White race *- n (%)*	235 (58.8)	87 (54.0)	148 (61.9)	0.10
Non-hispanic ethnicity	390 (97.5)	158 (98.1)	232 (97.1)	0.88
*Comorbidities*				
HFrEF *- n (%)*	259 (64.8)	94 (58.4)	165 (69.0)	0.03
Coronary artery disease *- n (%)*	163 (40.8)	58 (36.0)	105 (43.9)	0.12
Atrial fibrillation/flutter *- n (%)*	114 (28.5)	40 (24.8)	74 (31.0)	0.19
CKD (any stage) *- n (%)*	90 (22.5)	33 (20.5)	57 (23.8)	0.43
VT/VF *- n (%)*	85 (21.3)	28 (17.4)	57 (23.8)	0.12
*Hospitalization data*				
Pre-tMCS cardiac arrest *- n (%)*	61 (15.3)	36 (22.4)	25 (10.5)	<0.01
*Lactate	3 (4.75)	3.45 (5.78)	2.5 (3.20)	<0.01
*ALT	56.5 (195)	74.5 (301)	50 (112)	0.04
*Creatinine	1.7 (1.30)	1.76 (1.73)	1.7 (1.20)	0.55
LVEF by Echo	20 (14)	20 (15)	21 (13)	0.96
*Device data*				
First device				
Femoral IABP *- n (%)*	142 (35.5)	49 (30.4)	93 (39.0)	<0.01
Femoral Impella CP *- n (%)*	129 (32.3)	71 (44.1)	58 (24.3)	<0.01
Axillary IABP *- n (%)*	53 (13.3)	15 (9.3)	38 (15.9)	0.41
Axillary Impella 5.5 *- n (%)*	76 (19.0)	26 (16.1)	50 (21.0)	0.97
Any device exposure				
Femoral IABP - *n (%)*	151 (37.8)	55 (34.2)	96 (40.2)	0.25
Femoral Impella CP - *n (%)*	152 (38.0)	90 (55.9)	62 (26.0)	<0.01
Axillary IABP - *n (%)*	66 (16.5)	21 (13.0)	45 (18.8)	0.13
Axillary Impella 5.5 - *n (%)*	120 (30.0)	49 (30.4)	71 (29.7)	0.91
VA-ECMO *- n (%)*	68 (17.0)	47 (29.2)	21 (8.8)	<0.01
*CSWG-SCAI stage*				< 0.01
Stage B or C *- n (%)*	183 (46.3)	56 (35.4)	127 (53.6)	
Stage D or E *- n (%)*	212 (53.7)	102 (64.6)	110 (46.4)	
*Cardiomyopathy etiology*				0.94
Non-Ischemic *- n (%)*	208 (52.5)	81 (51.3)	127 (53.4)	
Ischemic *- n (%)*	178 (44.9)	73 (46.2)	105 (44.1)	
Mixed *- n (%)*	10 (2.5)	4 (2.5)	6 (2.5)	
*CS Etiology*				0.04
Acute on chronic HF/other *- n (%)*	269 (67.3)	98 (60.9)	171 (71.5)	
Acute MI or De-Novo *- n (%)*	128 (32.0)	61 (37.9)	67 (28.0)	
**Hemodynamic data				
Mean arterial pressure, mmHg	79 (17) *n* = 294	80.52 (25) *n* = 103	80 (14) *n* = 191	0.66
Right atrial pressure; mmHg	15 (10) *n* = 301	15 (10) *n* = 106	14 (10) *n* = 195	0.31
PCWP; mmHg	25.5 (12) *n* = 300	25 (11) *n* = 106	26 (12) *n* = 194	0.89
PAPi	1.5 (1.59) *n* = 297	1.41 (1.57) *n* = 105	1.54 (1.46) *n* = 192	0.15
Cardiac output; L/min	3.21 (1.29) *n* = 279	3.15 (1.48) *n* = 89	3.23 (1.19) *n* = 190	0.85
Cardiac index; L/min/m^2^	1.6 (0.51) *n* = 279	1.57 (0.52) *n* = 89	1.6 (0.51) *n* = 190	0.95
Cardiac power output; W	0.56 (0.27) *n* = 270	0.53 (0.32) *n* = 86	0.57 (0.26) *n* = 184	0.86

Data expressed as median (interquartile range) unless otherwise noted.

ALT, alanine aminotransferase; CKD, chronic kidney disease; CSWG, Cardiogenic Shock Working Group; DRAE, device-related adverse event; HF, heart failure; HFrEF, heart failure with reduced ejection fraction; IABP, intra-aortic balloon pump; LVEF, left ventricular ejection fraction; MI, myocardial infarction; PAPi, pulmonary artery pulsatility index; PCWP, pulmonary capillary wedge pressure; SCAI, Society for Cardiovascular Angiography and Interventions; tMCS, temporary mechanical circulatory support; VT/VF, ventricular tachycardia/ventricular fibrillation.

* Peak value within 24 hours of first device placement.

** Invasive hemodynamic measurements collected prior to the first tMCS device implantation.

**Figure 1:**
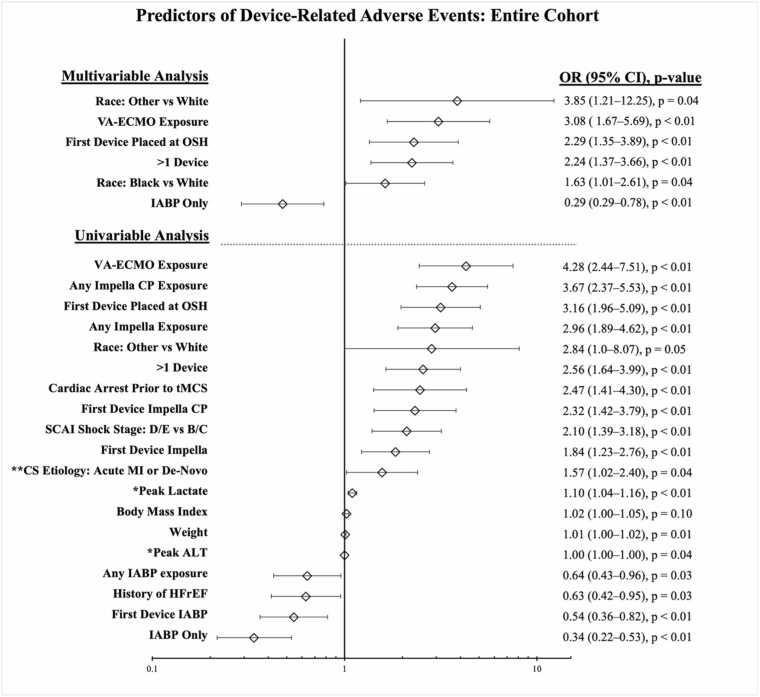
*Forest plot demonstrating uni- and multivariable predictors of device-related adverse events in patients with intra-aortic balloon pump and/or microaxial flow pump for cardiogenic shock.* *Peak values represent the highest value within 24 hours of first device placement. ALT, alanine aminotransferase; CI, confidence interval; CS, cardiogenic shock; DRAEs, device-related adverse events; HFrEF, heart failure with reduced ejection fraction; IABP, intra-aortic balloon pump; MI, myocardial infarction; OR, odds ratio; OSH, outside hospital; SCAI, Society for Cardiovascular Angiography and Interventions; tMCS, temporary mechanical circulatory support; VA-ECMO, veno-arterial extracorporeal membrane oxygenation.

Univariable analysis in the overall population yielded 19 variables with *p* < 0.1 (Figure 1). The strongest DRAE predictors were VA-ECMO exposure and Impella CP exposure. Protective variables included IABP only and first device IABP. Multivariable analysis yielded 4 independent predictors of DRAEs (race [other vs white; black vs white], VA ECMO exposure, initial device placement at outside hospital [OSH], and >1 device) and 1 protective variable (IABP only). The final model demonstrated good fit (χ^2^ = 3.17; *p* = 0.92) and explained 22.2% of the outcome variance (pseudo-R^2^ = 0.22).

**Figure 2:**
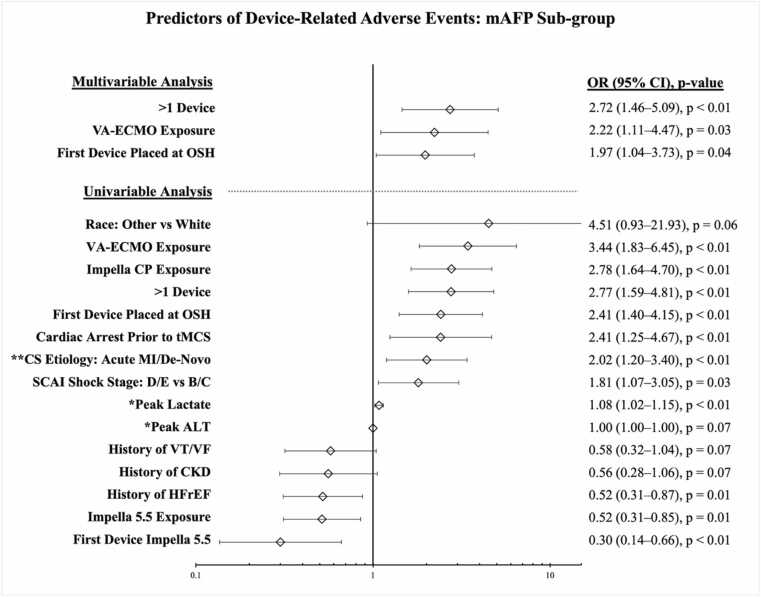
*Forest plot demonstrating uni- and multivariable predictors of device-related adverse events in patients with microaxial flow pump for cardiogenic shock.* *Peak values represent the highest value within 24 hours of first device placement. ALT, alanine aminotransferase; CI, confidence interval; CKD, chronic kidney disease; CS, cardiogenic shock; DRAEs, device-related adverse events; HFrEF, heart failure with reduced ejection fraction; IABP, intra-aortic balloon pump; MI, myocardial infarction; OR, odds ratio; OSH, outside hospital; SCAI, Society for Cardiovascular Angiography and Interventions; tMCS, temporary mechanical circulatory support; VA-ECMO, veno-arterial extracorporeal membrane oxygenation; VT/VF, ventricular tachycardia/ventricular fibrillation.

Univariable analysis in the mAFP sub-group yielded 15 variables with *p* < 0.1 (Figure 2). The strongest predictive variables were VA-ECMO exposure, Impella CP exposure, and >1 device. Protective variables included Impella 5.5 as the first device and any Impella 5.5 exposure. Multivariable analysis yielded 3 independent DRAE predictors (>1 device, VA-ECMO exposure, and initial device placement at OSH). The final model demonstrated good fit (χ^2^ = 3.53; *p* = 0.74) and explained 18.6% of the outcome variance (pseudo-R^2^ = 0.19).

## Discussion

In this analysis, DRAEs occurred in 40% of patients receiving IABP or mAFP and were primarily associated with device exposure (VA-ECMO exposure, OSH device placement, and multiple devices). Non-white race was the only independent DRAE predictor unrelated to device exposure.

This analysis offers novel insight into risks of tMCS in a contemporary CS population. First, adjudicated adverse events (AEs) were specifically attributable to tMCS use. Prior analyses describing AEs in patients with CS and tMCS^5^ lack granularity to assess temporal correlation between AEs and tMCS. Second, randomized trials describing AEs in tMCS-treated populations have focused on myocardial infarction-related CS,^6^, ^7^ which represents only ∼30% of patients with CS.^8^ CS etiology in our analysis more closely represents a contemporary population (67% with acute-on-chronic heart failure CS). Lastly, 28.3% of patients in our analysis were exposed to >1 tMCS device, which is common in contemporary practice and is an independent predictor of DRAEs. Efforts should be made to reduce multi-device exposure, possibly selecting up-front high-capacity tMCS devices rather than bridging with lower-capacity devices.

Most DRAE predictors in our this analysis were related to tMCS exposure. Non-white race was the sole independent DRAE predictor unrelated to tMCS exposure. Racial diversity is a strength of our study population. Although non-white race is a known predictor of complications among patients with CS^9^ and those with tMCS for acute myocardial infarction,^10^ race has not been investigated in a contemporary population with tMCS for CS. While clinical factors such as body size and creatinine have been the focus of patient selection and tMCS management strategies, investigation into mechanisms underlying race-associated DRAE risk is warranted to ensure equitable care.

Limitations of this analysis include the single-center retrospective design, risk of misclassification and unmeasured confounding, and variable collinearity, though the latter was accounted for statistically. There was missingness in some hemodynamic variables, particularly in patients with OSH-implanted devices, which precluded inclusion of hemodynamics into the multivariable analysis. The use of a single composite DRAE outcome was necessary for multivariable regression due to low individual DRAE counts, which limited our ability to identify predictors of specific DRAEs.

In conclusion, we found that tMCS exposure is the strongest driver of DRAE risk; however, non-white race represents an underrecognized DRAE predictor. Further investigation is warranted to explore the association of non-white race with DRAEs and implement risk mitigation strategies.

## Disclosure statement

Dodson reports consulting for Johnson & Johnson MedTech; Tedford reports no disclosures relevant to this manuscript. He has served on a research advisory board for Abiomed. He reports general disclosures to include consulting relationships with and receiving honorarium/consulting fees from Abbott, Acorai, Adona, Aria CV Inc., Acceleron, Boston Scientific, CVRx, Endotronix, Edwards LifeSciences, Fauna Bio, Gradient, Imbria, Medtronic, Merck, Morphic Therapeutics, Pulmovant, Restore Medical, Tempus AI, and United Therapeutics. Dr Tedford serves on steering committees for Abbott, Edwards, Endotronix, Gradient, Merck and Tempus AI; Kilic reports no disclosures relevant to this manuscript. He reports general disclosures to include consulting relationships with and receiving honorarium/consulting fees from Abbott, 3ive, and LivaNova. He is a founder/owner of QImetrix; Houston reports general disclosures to include consulting relationships with Abbott, Edwards Lifesciences, Gradient, and Aria CV; Carnicelli reports research support from Acorai and Johnson & Johnson MedTech paid to the institution, speaker honoraria and travel support from Johnson & Johnson MedTech, and consulting fees from Precision Cardiovascular; all other authors report no relevant disclosures.

## Conflicts of interest statement

The authors declare that they have no known competing financial interests or personal relationships that could have appeared to influence the work reported in this paper.

## Footnotes

Appendix A Supplementary data associated with this article can be found in the online version at doi:10.1016/j.jhlto.2026.100557.
