# Supplementary material for: Predictors of device-related adverse events in patients with intra-aortic ballon pump or microaxial flow pump for cardiogenic shock
Source: JHLT Open. 2026 Apr 8;13:100557. doi: 10.1016/j.jhlto.2026.100557 (PMC13129373; doi:10.1016/j.jhlto.2026.100557)
Supplement: Supplementary file 1 — Supplementary material [file mmc1.docx]

**Supplement Table S1.** Adverse event definitions adapted from the Cardiogenic Shock Academic Research Consortium Standardized Definitions for Cardiogenic Shock Research and Mechanical Circulatory Support Devices.

| **Adverse Event** | **Standardized Definition** |
| --- | --- |
| **Bacteremia** | Two positive blood cultures drawn at the same time with no other source identified requiring treatment with intravenous antibiotic therapy |
| **Bleeding** | A drop in hemoglobin ≥4 g/dL or bleeding requiring transfusion of ≥2 units of packed red blood cells in a single day, excluding bleeding during/after durable left ventricular assist device implantation or heart transplantation |
| **Hemolysis** | Increase in serum lactate dehydrogenase and plasma free hemoglobin and a change in urine color |
| **Heparin Induced Thrombocytopenia** | Thrombocytopenia with documented positive PF4 testing and positive serotonin release assay |
| **Stroke** | Acute onset of focal neurological signs or symptoms lasting >24 hours and radiographic evidence confirming acute ischemic or hemorrhagic stroke conforming to a corresponding vascular territory |
| **Transient Ischemic Attack** | Transient focal neurological signs or symptoms lasting <24 h presumed to be due to focal brain, spinal cord, or retinal ischemia, but without evidence of acute infarction by neuroimaging or pathology, or with no imaging performed |
| **Vascular Injury** | Vascular (arterial or venous) injury (perforation, rupture, dissection, stenosis, ischemia, thrombosis, embolism, arteriovenous fistula, pseudoaneurysm, hematoma, retroperitoneal hematoma) diagnosed radiographically resulting in some clinical manifestation (bleeding, ischemia, neurologic impairment) or treated with vascular intervention |

**Supplement Table S2.** Device-related adverse event incidence in the overall study population and by device exposure.

| **Device Related Adverse Event Incidence** | | | |
| --- | --- | --- | --- |
| **Adverse Event** | **Study Population (n=400)** | **Any IABP Exposure (n=201)** | **Any mAFP Exposure (n=251)** |
| **Significant Bleeding** | 64 (16.0%) | 30 (14.9%) | 53 (21.1%) |
| **Major Hemolysis** | N/A | N/A | 51 (20.3%) |
| **Bacteremia** | 44 (11.0%) | 23 (11.4%) | 28 (11.2%) |
| **Vascular Injury** | 35 (8.8%) | 16 (8.0%) | 28 (11.2%) |
| **Heparin Induced Thrombocytopenia** | 13 (3.3%) | 5 (2.5%) | 8 (3.2%) |
| **Stroke/TIA** | 11 (2.8%) | 7 (3.5%) | 9 (3.6%) |

**Supplement Figure S1.** Sankey diagram depicting sequence of 1^st^ and 2^nd^ devices among 113 patients who received >1 temporary mechanical circulatory support device.

**
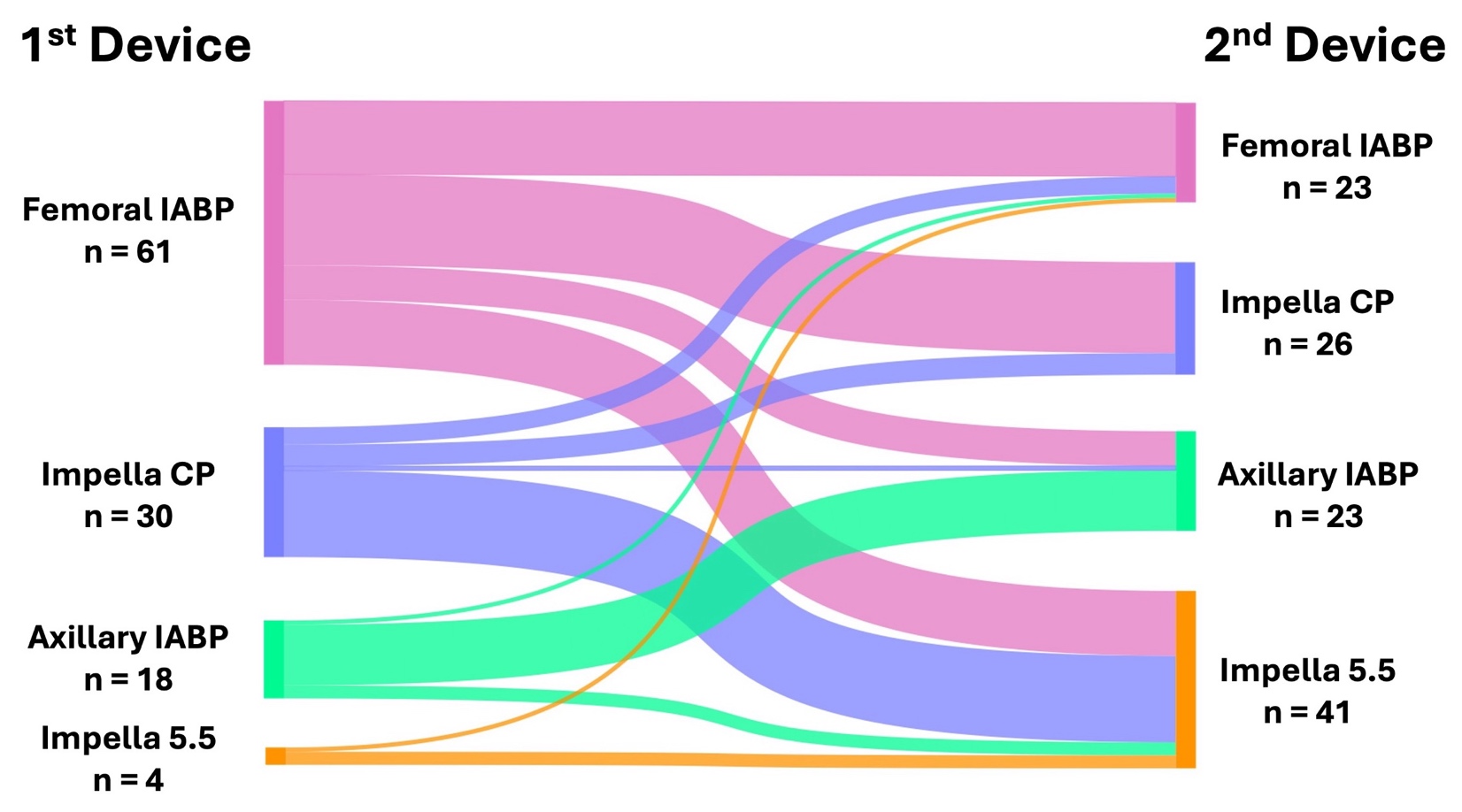
**

IABP = intra-aortic balloon pump
